# Tetraazacyclohexadeca Macrocyclic Ligand as a Neutral Carrier in a Cr Ion-selective Electrode

**DOI:** 10.3390/s041200187

**Published:** 2004-12-22

**Authors:** Ashok Kumar Singh, Rupam Singh, Puja Saxena

**Affiliations:** 1 Department of Chemistry, Indian Institute of Technology-Roorkee, Roorkee - 247 667, India

**Keywords:** chromium-selective electrode, macrocyclic ionophore, potentiometric sensor

## Abstract

A polystyrene-based membrane of 2,10-dimethyl-4,12-diphenyl-1,5,9,13-tetraazacyclohexadeca-1,4,9,12-tetraene macrocyclic ionophore was prepared and investigated as Cr(III)-selective electrode. The best performance was observed with the membrane having the polystyrene-ligand-dibutylphthalate-sodiumtetraphenyl borate composition 1:4:1:1 with a Nernstian slope of 19.0 mV per decade of concentration between pH 3.0 and 6.5. This electrode has been found to be chemically inert and of adequate stability with a response time of 20 s and was used over a period of 100 d with good reproducibility (S= 0.3 mV). The membrane works satisfactorily in a partially non-aqueous medium up to a maximum 30% (v/v) content of methanol and ethanol. The potentiometric selectivity coefficient values indicate that the membrane sensor is highly selective for Cr(III) ions over a number of monovalent, divalent and trivalent cations. The membrane electrode has also been successfully used to determine Cr^3+^ in various food materials.

## Introduction

The environmental and health effects of metal ion pollution are important and complex problems. From the broad point of view, almost every metal or metallic compound encountered in manufacturing industry present some type of ecological hazard. From the more realistic point of view, the principal health hazards are caused by lead, mercury, arsenic, chromium, cadmium, antimony and their compounds. Among these, chromium is a major pollutant. Hazards from chromates have been known for over 100 years. These occur generally in the form of ulcers known as “chrome holes”. The spray of chromic acid produced during the process of plating is injurious to workers. Practically, all of the chromium-plating baths now used have chromic acid as the principal component. Continuous daily exposure to concentrated chromic acid is likely to cause definite injury to the nasal tissues of the workers [1]. Contact with abrasions in the skin is also dangerous. Chromates also cause severe kidney damage and intestinal inflammation. That is why, from an environmental point of view, there is a strong need to develop new methods to determine chromium (III) ions in chemical and biological samples. Ionophore-based membrane electrodes are well-established analytical tools, routinely used for the measurement of a wide variety of different ions directly in complex biological and environmental samples [2-5]. In this respect, macrocycles have been widely used as suitable neutral carriers for constructing membrane selective electrodes for heavy and toxic metals [6-9].

Very little work has been done on the development of ion-selective electrodes (ISEs) for Cr(III) ions. The first report on Cr(III) appeared in 1980 [10]. In this report, a PVC-based chromium (III)-wire electrode was made by incorporating (Aliquat 336S^+^-Cr(SCN)_4_^-^) ion pair, and the electrode gave a fully linear response of 10^-5^-10^-2^ mol L^-1^and has the Nernstian slope of 58.0 mV per decade of concentration. In 1989, an ion-selective electrode based on chromium dithizonate was built, that was a precipitate-based selective electrode [11]. In addition to these, chromium-selective electrodes based on nickel tris (1,10-bathophenanthroline) hydrogen chromate [12], 2,4,9,11-tetraphenyl-1,5,8,12-tetraazacyclotetradeca-1,4,8,11-tetraene dihydrogen perchlorate [13], 4-dimethylaminoazobenzene [14], 3,10-c-*meso*-3,5,7,7,10,12,14,14-octamethyl-1,4,8,11-tetraazacyclotetradecane diperchlorate [15] and glyoxal bis(2-hydroxyanil) [16] ionophores have also been determined. Some of the recent electrodes have been compared to the proposed electrode assembly which shows that the electrode presented in this paper has a Nernstian response with a wide working concentration range and fast response time than the earlier reported electrodes (Table 1).

## Experimental

All the reagents and chemical used were of analytical grade. Double distilled water was used for the preparation of solutions of metal salts of different concentration by dilution of stock standard solutions (0.1 M). Polystyrene was obtained from G.S.C. (New Delhi, India). Chromium chloride was used as a source of Cr(III) ions for the studies of the membrane sensor.

### Synthesis of macrocycle

**Scheme 1.**
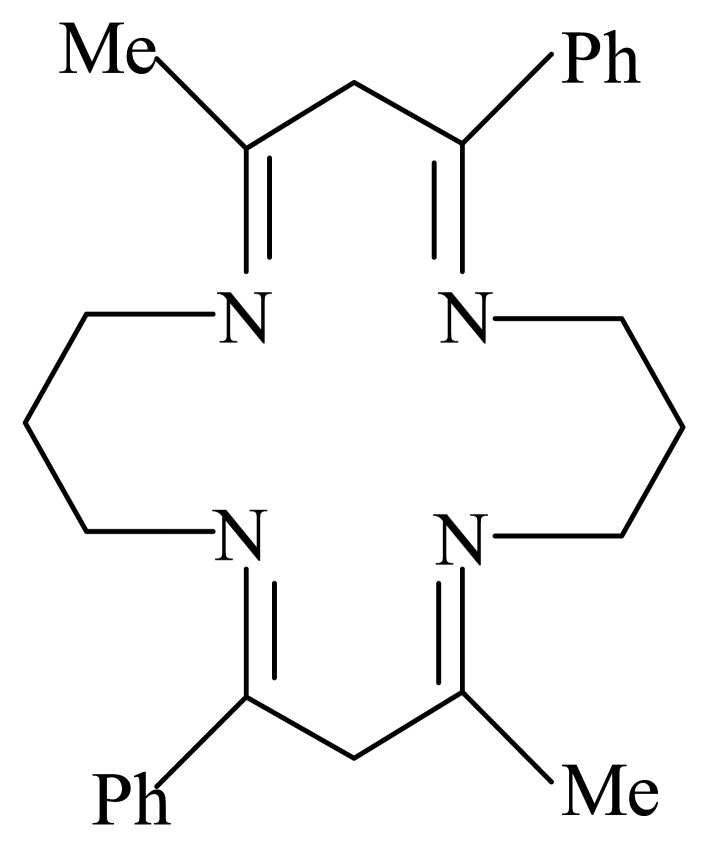
2,10-dimethyl-4,12-diphenyl-1,5,9,13-tetraazacyclohexadeca-1,4,9,12-tetraene.

1.67 mL (0.02 mol) of 1,3-diaminopropane was dissolved in 50 mL of methanol and cooled in ice. To this, a solution of 3.24 g (0.02 mol) of 1-phenyl-1,3-butanedione in 50 mL methanol was added. The solution was stirred for 24 hours at 5 °C, refluxed for 8 hours and then concentrated to about 20 ml under vacuum and placed in an ice bath. The white colored product was washed with ice-cold methanol and recrystallised twice from methanol-water mixture [C_26_H_32_N_4_-calculated (%): C, 77.96; H, 8.05; N, 13.99, found (%): C, 78.12; H, 8.25; N, 13.96]. The calculated and observed elemental analysis data for the ligand are in good agreement with the structure (see scheme 1).

### Electrode preparation

A number of membranes [17-19] were prepared to have one of adequate strength with a minimum amount of binder (polystyrene) with excellent reproducibility, stability and fast response time.

To satisfy these requirements, varying (Table 2) ratios of macrocycle *vs.* polystyrene were taken and the mixture was heated to 80 °C (softening point of polystyrene) under pressure (6000 to 6500 psi). Membranes prepared in this way were quite stable and did not show any dispersion in water and in other electrolyte solutions.

The membranes were also subjected to microscopic and electrochemical examination for cracks and homogeneity of the surface and only those which had a smooth surface and generated reproducible potentials were chosen for the subsequent investigation. Membrane to membrane (and batch to batch) reproducibility were ensured by carefully controlling the condition of fabrication. The membranes (2.5 cm diameter and 0.5 cm thick) were affixed to one end of a small pyrex glass tube with epoxy resin adhesive (Aradite), while the other remained open.

### Potential measurements

Membranes were equilibrated with 1.0 mol L^-1^ Cr(III) chloride solution for 6 days and the potential across the membrane was measured with an ECIL (Hyderabad, India) digital pH meter potentiometer Model pH 5662, and Century (Chandigarh, India) CBM 301 microvoltameter in conjunction with saturated calomel electrodes (SCE) as reference electrodes. The Debye-Huckel procedure was used for the activity concentration relation [20].

Response times were determined after the potential of one chromium solution had become constant, and similar measurements were carried out in another solution of 100-fold lower concentration. The response time is defined as the time taken to reach a potential of 90% of the potential difference in the two measurements. Reproducibility was defined by the deviation from the average potential value in the same four ‘dip to read’ measurements.

### Sample preparation and determination of Cr(III) in various food materials

Samples of tea leaves and turmeric powder for the determination of Cr were prepared by wet ashing and for dehydrogenated vegetable oil by dry ashing [21]. The solutions were analyzed using an AAS 6500 Atomic Absorption Spectrometer (Perkin-Elmer, Nor Walk, CT, USA) and also using the sensor developed, here after adjusting the pH of the sample. The results reported are the averages of a minimum of three determinations.

## Results and discussion

To assure the use of a particular ionophore as a Cr(III) ion-selective electrode, a number of membranes with 25% polystyrene were equilibrated for various monovalent, bivalent and trivalent cations. The results drawn from the cell potentials showed that the response to cations other than Cr^3+^ was non-Nernstian with a poor working concentration range, whereas as for Cr^3+^ the response was Nernstian. Hence, the ionophore was used for further studies as Cr^3+^-ISE.

The optimum conditions for the best performance of the Cr(III)-ISE based on a macrocyclic ligand membrane were investigated systematically.

### Working concentration range

**Figure 1.**
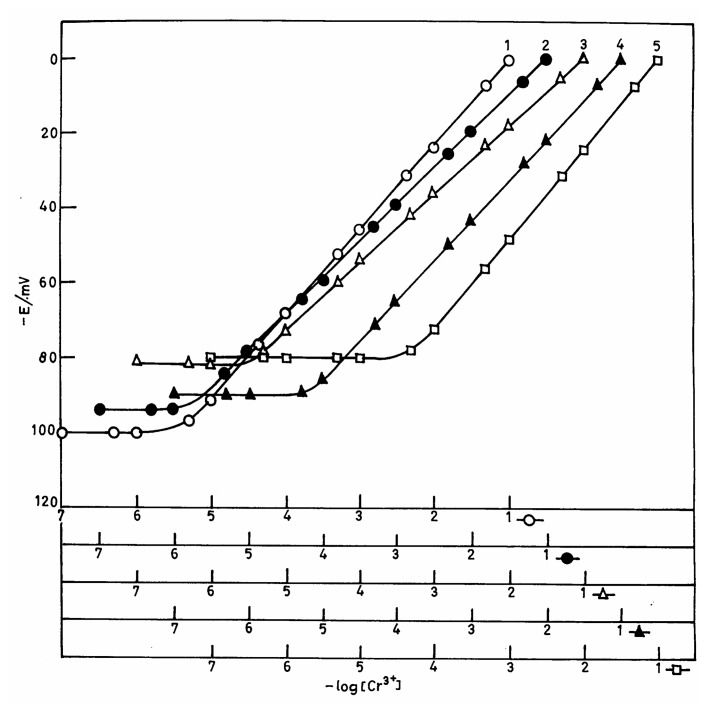
Plots showing the variation of the membrane potential of different macrocyclic ligands with the concentration of Cr(III) ions. The numbers on the plots refer to compositions given in Table 2.

The membrane was equilibrated with 1.0 mol L^-1^ chromium(III) chloride solution for three days. The potentials generated after three days were stable and reproducible. The working concentration ranges and slopes for all the five membranes are given in Table 2. It was observed that the membrane No. 2 with polystyrene and ionophore, dibutylphthalate (plasticizer) and sodium tetraphenylborate (anion excluder) in 1:4:1:1 ratio (w/w) had the widest concentration range of 1.6×10 ^6^-1.0×10^1^ mol L^-1^ and a near-Nernstian slope of 19.5 mV/decade. Membrane No. 1 with ionophore and polystyrene in 2:1 ratio (w/w) showed a non-Nernstian slope of 22.5 mV/decade of concentration. Similarly, when the polystyrene ratio was decreased, the response was also non-Nernstian and the working concentration range becomes poorer (3.2×10^-6^ -1.0×10^-1^ mol L^-1^ for membrane No. 3, 7.1×10^-6^ -1.0×10^-1^ mol L^-1^ for membrane No. 4 and 5.0×10^-5^-1.0×10^-1^mol L^-1^ for membrane No. 5, see also Fig 1). Hence, the membrane No. 2 was studied in detail as a Cr(III)-selective electrode and all further investigations were carried out with this particular membrane.

### Response and life time

The membrane No. 2 with 25% of polystyrene observed the fastest response time of 20 s and a wide working concentration range of 1.6×10^-6^-1.0×10^-1^ mol L^-1^. As the amount of polystyrene decreased (membrane No. 3 and 4), the response time became poorer (>25 s). Similarly, when the percentage of polystyrene was increased (membrane No. 1), the response time was 25 s (Table 2). The potential so obtained remained constant for more than 15 min, after which a slow divergence was observed.

The membrane electrode was used over a period of 100 days without any significant change in potentials and repeated monitoring of potentials at a fixed concentration gave a standard deviation of 0.3 mV. When not in use the membranes were stored in 1.0×10^-1^ mol L^-1^ Cr^3+^ solution and whenever any drift in potentials was observed, the membranes were re-equilibrated with 1.0 mol L^-1^ Cr^3+^ solution for 3-4 days. The electrochemical behavior of the electrode gradually deteriorated after 100 days, which can be attributed to the loss of electrically neutral ionophores into the sample [22].

### pH and solvent effect

**Figure 2.**
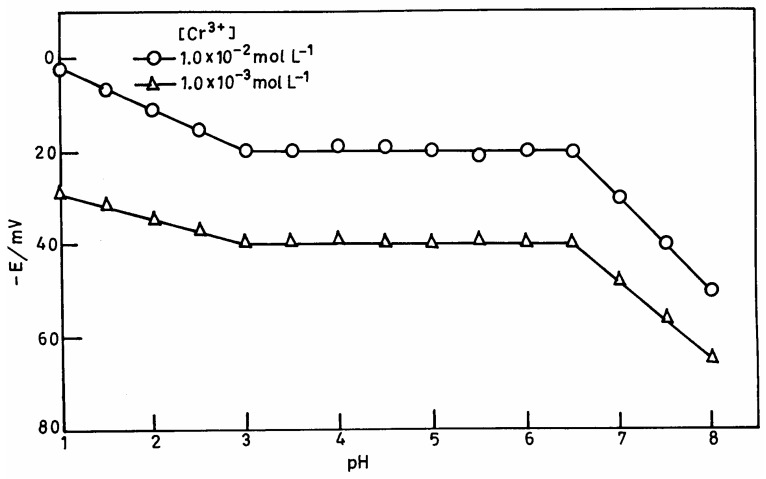
The variation of membrane potential with pH at 1.0×10^-2^ mol L^-1^ and 1.0×10^-3^ mol L^-1^ Cr^3+^.

The pH dependence of electrode potential was tested over a pH range 1.0-8.0 for 1.0×10^-2^ mol L^-1^ and 1.0×10^-3^ mol L^-1^ of Cr^3+^. The pH was adjusted with nitric acid or ammonia solution. The potential was independent in the range 3.0-6.5 (Figure 2) and the same can be taken as the working pH range of the electrode assembly. The change in potentials above pH 6.5 may be due to the hydrolysis of Cr^3+^ and below 3.0 due to a H^+^ ion interference.

**Figure 3.**
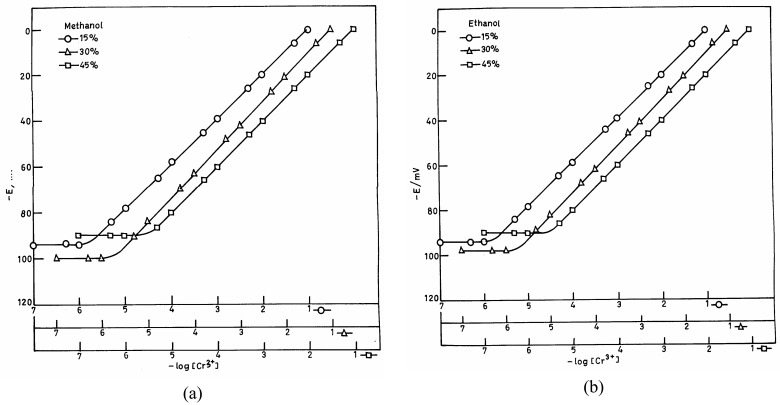
Plots of potential vs. log [Cr(III)] of mixed solutions: (a) water-methanol and (b) water-ethanol.

The practical utility of the proposed sensor was investigated in partially non-aqueous media using 15%, 30% and 45% water-methanol and water-ethanol mixtures. Figures 3 (a) and (b) indicate a reduction in the linear portion of the potential *vs.* concentration plot in non-aqueous media. This decrease is however nominal when the non-aqueous content is 30%, but a further increase in non-aqueous content causes a significant interference. Hence, the electrode assembly can only be used in a non-aqueous medium when its content is not more than 30%.

### Selectivity

Potentiometric selectivity coefficients were determined by the matched potential method [23] at a 1.0×10^-2^ mol L^-1^ concentration of interfering ions. The selectivity coefficients given in Table 3 indicate a good selectivity over monovalent, divalent and trivalent cations. Hence, these are not expected to interfere even at a high concentration level (1.0×10^-2^ mol L^-1^) of interfering ions. Further, to investigate the effect of anions, cell potentials were obtained using chromium sulphate. No significant changes in the working concentration range and slope were observed, indicating that these anions (Cl^-^ and SO_4_^2-^) do not cause any interference.

### Estimation of Cr(III) in various food materials

The electrode has been successfully applied for the direct determination of Cr^3+^ in various food materials. No other treatment of the sample was necessary except a pH adjustment. The pH for all samples was adjusted at 5.0. The reports on the results obtained using this electrode showed a good agreement.

## Conclusions

A membrane sensor incorporating 2,10-dimethyl-4,12-diphenyl-1,5,9,13-tetraazacyclohexadeca-1,4,9,12-tetraene can estimate Cr(III) ions in the concentration range 1.6 × 10^-6^-1.0×10^-1^ mol L^-1^ with a slope of 19.5 mV per decade of activity. This electrode (No. 2) is chemically stable and gives reproducible results with a useful lifetime of 100 days, exhibiting a Nernstian slope within a functional pH range 3.0-6.5. The electrodes can also be used successfully in non-aqueous media. Above all, the proposed sensor was successfully applied to the estimation of Cr(III) in real samples.

## Figures and Tables

**Table 1.** Comparative studies of previously reported literature based on Cr^3+^ ion-selective electrode.

**No.**	**Ionophore**	**Working conc. range/M**	**Slope/mV/dec.**	**pH Range**	**Response time/s**	**Ref.**
1	Nickel ris(1,10-batho-phenanthroline) hydrogen chromate	2.0×10^-2^-8.0×10^-6^	55.5	5.0	-	[11]
2	Macrocycle based	1.8×10^-6^-1.0×10^-1^	20.0	3.0-6.5	15	[12]
3	4-Dimethyl-aminoazobenzene	1.7×10^-6^-1.0×10^-2^	19.5	3.0-5.5	10	[13]
4	Glyoxal bis(2-hydroxyanil)	4×10^-6^-1.0×10^-1^	19.8	2.7-6.5	<20	[15]
5	Tetraaza macrocyclic based	1.6×10^-6^-1.0×10^-1^	19.5	3.0-6.5	18	Present Electrode

**Table 2.** Composition of polystyrene based membranes of [Me_2_Ph_2_(16)tetraene N_4_] and their performance as Cr^3+^ selective electrode.

**Mem-braneNo.**	**Composition of Membrane (proportions)**				**Working Concentration Range/mol L^-1^**	**Slope/mV/dec.**	**Response Time/s**

	**Iono-phore**	**Poly-styrene**	**DBP**	**STB**			
1.	2	1	1	1	4.0×10^-6^-1.0×10^-1^	22.5	25
2.	4	1	1	1	1.6×10^-6^-1.0×10^-1^	19.5	18
3.	6	1	1	1	3.2×10^-6^-1.0×10^-1^	18.5	28
4.	8	1	1	1	7.1×10^-6^-1.0×10^-1^	21.5	30
5.	10	1	-	1	5.0×10^-5^-1.0×10^-1^	24.0	20

**Table 3.** Selectivity coefficient values for a Cr(III)-selective membrane electrode based on [Me_2_Ph_2_(16)tetraene N_4_] Cr(III) macrocyclic complex.

**Interferent (B)**	Selectivity coefficient $\left(K_{cr^{3+},B}^{\text{pot}}\right)$	**Interferent (B)**	Selectivity coefficient $\left(K_{cr^{3+},B}^{\text{pot}}\right)$
Li^+^	1.80 × 10^-1^	Cu^2+^	2.66 × 10^-2^
Na^+^	1.75 × 10^-1^	Ni^2+^	3.26 × 10^-3^
Ag^+^	4.42 × 10^-3^	Zn^2+^	2.25 × 10^-1^
Ca^2+^	1.41 × 10^-2^	Mg^2+^	2.38 × 10^-2^
Ba^2+^	8.92 × 10^-2^	Fe^3+^	2.25 × 10^-4^
Hg^2+^	7.00 × 10^-3^	Al^3+^	7.08 × 10^-4^
Sr^2+^	7.50 × 10^-2^	Bi^3+^	3.82 × 10^-4^
Pb^2+^	1.42 × 10^-3^	La^3+^	2.66 × 10^-5^
Cd^2+^	6.80 × 10^-3^		
